# Research progress of megakaryocytes and platelets in lung injury

**DOI:** 10.1080/07853890.2024.2362871

**Published:** 2024-06-20

**Authors:** Tianzhen Hua, Guangliang Zhang, Yi Yao, Haoran Jia, Wei Liu

**Affiliations:** a Department of Burns and Plastic Surgery, The Fourth Medical Center, Chinese PLA General Hospital, Beijing, China; b Medical School of Chinese PLA, Beijing, China

**Keywords:** Megakaryocytes, platelets, acute lung injury, chronic lung injury, acute respiratory distress syndrome

## Abstract

The lung is an important site of extramedullary platelet formation, and megakaryocytes in the lung participate in immune responses in addition to platelet production. In acute lung injury and chronic lung injury, megakaryocytes and platelets play a promoting or protective role through different mechanisms. The authors reviewed the role of megakaryocytes and platelets in common clinical lung injuries with different course of disease and different pathogenic factors in order to provide new thinking for the diagnosis and treatment of lung injuries.

## PLAIN LANGUAGE SUMMARY

What is the context?

Platelets are specialized non-nucleated blood cells produced by cytoplasmic lysis of megakaryocytes.

HSCs differentiate into granular mature megakaryocytes and produce platelets.

Lung is a reservoir of megakaryocytes and a site where platelets are produced in addition to bone marrow and spleen.

Lung injury can be divided into acute lung injury and chronic lung injury, and characterized by different pathogenesis.

Platelets and megakaryocytes are involved in hemostasis and regulation of the body ‘s inflammatory response.

The disease state of the lung affects the functions of megakaryocytes and platelets.

The role of megakaryocytes and platelets in acute and chronic lung injury is poorly studied.

What is new?

Platelets in the lung are derived not only from the spleen and bone marrow, but also from megakaryocytes in the pulmonary circulation. In this study, we demonstrated that pulmonary megakaryocytes not only produce platelets to play a hemostatic role in lung injury, but also participate in inflammation and immune response with platelets to promote the process of lung injury or play a protective role.

Therefore, it was suggested in our analysis that targeting lung megakaryocytes and platelets is currently a new direction for the treatment of a variety of lung injuries.

What is the impact?

This review intends to explain the relationship between megakaryocytes, platelets and many types of lung injury from the mechanism of platelet production in the lung, and make a prospect in the new progress in the diagnosis and treatment of lung injury.

## 1. Introduction

Platelets are specialized non-nucleated blood cells unique to mammals and are produced by cytoplasmic lysis of megakaryocytes. The origin of megakaryocytes is hematopoietic stem cells (HSCs) [1] from bone marrow. HSC is differentiated and matured into granular mature megakaryocytes that can produce platelets by megakaryocyte-erythroid progenitor (MEP) and immature megakaryocytes (including promegakaryoblasts, megakaryoblasts, and promegakaryocytes) under the action of regulators such as thrombopoietin (TPO), IL (Interleukin)-3, IL-6, and IL-11 [2]. Ultimately, granular megakaryocytes form proplatelets that protrude and explosively divide into platelets, or release platelets into the blood by forming platelet ribbons through pseudopodia [3]. Granular megakaryocytes derived from bone marrow reach the lung *via* the blood circulation. Lung is a reservoir of megakaryocytes and is recognized as one of the important sites of extramedullary hematopoiesis (EMH) where platelets are produced in addition to bone marrow and spleen [4]. Lung injury can be divided into acute lung injury and chronic lung injury according to different course of disease. And it can be divided into lung impact injury and lung penetrating injury caused by trauma, septic lung injury and pneumonic lung injury caused by infection, pulmonary ischemia and hypoxia and reperfusion injury caused by shock, inhalation injury caused by burn and secondary disseminated intravascular coagulation (DIC), etc [5]. according to different pathogenesis. Platelets are not only involved in hemostasis after lung injury, but are also implicated in the regulation of the body ‘s inflammatory response [6]. A study published in *Nature* in 2017 directly demonstrated megakaryocytes and the function of platelets produced in real-time by the mouse lung through lung intravital imaging, thus providing new ideas for the study of lung injury and lung-related diseases [4]. At the same time, the disease state of the lung also affects the related functions of megakaryocytes and platelets in the body. This review intends to explain the relationship between megakaryocytes, platelets and many types of lung injury from the mechanism of platelet production in the lung, and make a prospect in the new progress in the diagnosis and treatment of lung injury.

## 2. Mechanism of platelets production in the lung

### 2.1. Extrapulmonary megakaryocyte translocation and release of proplatelets

Platelets in the pulmonary circulation are formed from megakaryocytes and proplatelets released from bone marrow sinusoids and spleen [7]. In response to the effects of fibroblast growth factor-4 (FGF-4) and stromal cell-derived factor-1 (SDF-1), megakaryocytes in bone marrow and spleen translocate to the perivascular environment (e.g. sinusoids in bone marrow) and elongate the cytoplasm to form proplatelets [8]. Type I collagen in the extracellular matrix of bone marrow inhibits proplatelet formation *via* integrinα2β1 signaling [9] in mice; whereas von Willebrand factor (vWF) and fibrinogen in endothelial cells induce proplatelet formation. At the same time, proplatelets migrate transendothelially into the circulation through membrane and cytoskeleton reorganization and modification of microtubules and microfilaments, and under the action of circulatory shear stress and turbulence [10]. During this process, higher concentration of sphingosine-1 phosphate (S1P) in blood activates sphingosine −1 phosphate receptors on megakaryocytes, establishing a chemotactic gradient to direct proplatelet elongation and release into blood [11].

### 2.2. Formation of platelets in the pulmonary vascular system

**Figure 1.**
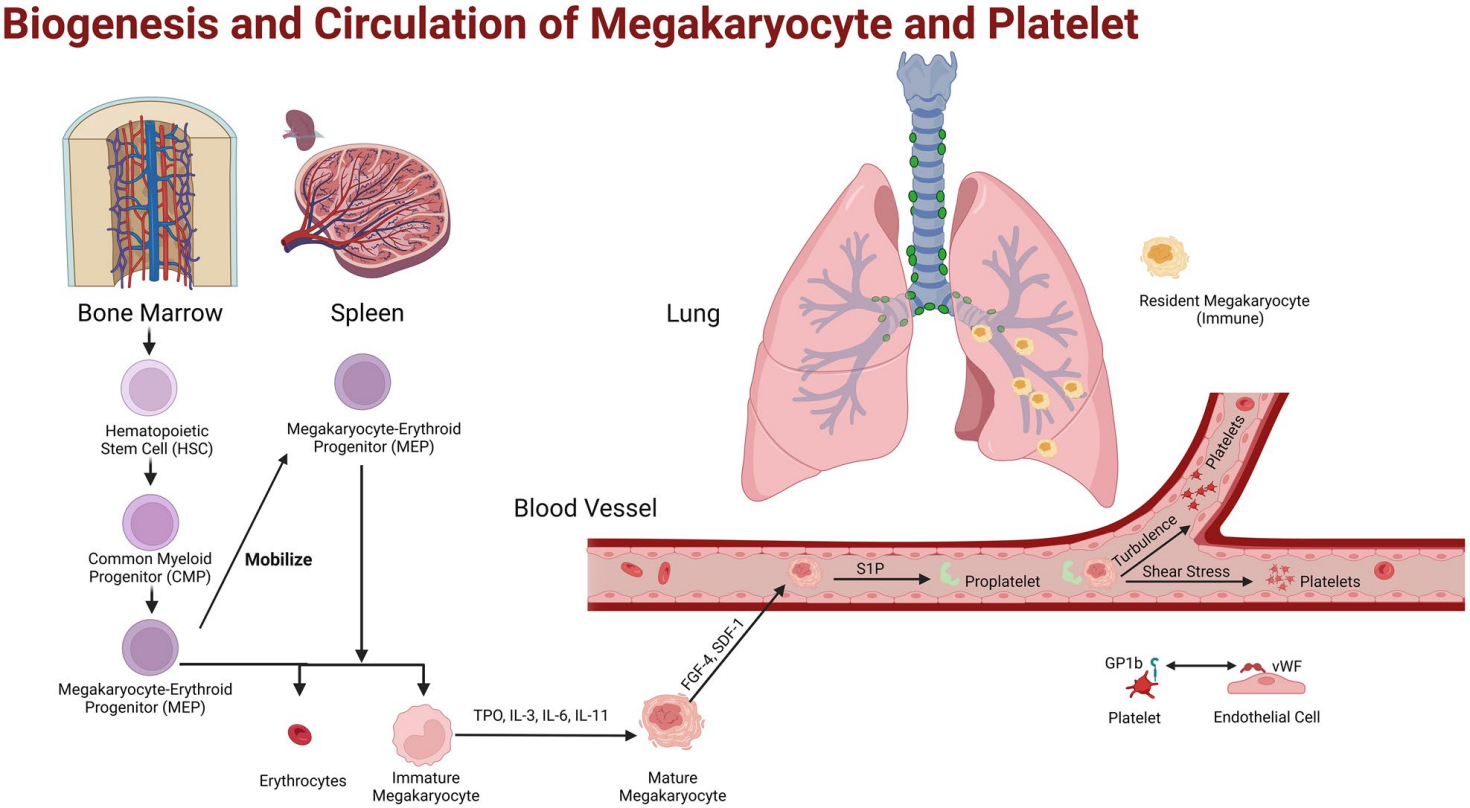
Biogenesis and Circulation of Megakaryocyte and Platelet. HSCs in bone marrow differentiate into megakaryocyte progenitors, which migrate to spleen, another site of hemopoiesis. Megakaryocytes mobilize into blood and release platelets in lung blood vessels through pulmonary circulation. Resident megakaryocytes in the pulmonary interstitium are found to have immunological properties.

In the bone marrow and spleen, platelet precursor cells, including elongated proplatelets, large protrusions of megakaryocytes, and intact megakaryocytes, can be released into the blood circulation. Intact megakaryocytes are between 40 μm and 70 μm in diameter, while capillaries in the lung are less than 5 μm in diameter, hindering the passage of platelet precursor cells [12] and providing conditions for platelet formation. On the one hand, megakaryocytes and proplatelets incarcerated in pulmonary capillaries undergo release of platelets in response to shear stress and turbulence provided by blood flow [13]. On the other hand, vWF expression is heterogeneous in the pulmonary vasculature, with high levels of vWF expression in pulmonary endothelial cells. The vWF receptors (Glycoprotein Ib, GPIb) expressed on megakaryocytes and platelets suggest this mechanism by which megakaryocytes regulate platelet production through interactions with lung endothelial cells [14] (Figure 1).

### 2.3. Types and effects of Megakaryocytes in the lung

The results of single-cell RNA sequencing analysis on fetal and adult mice model showed that megakaryocytes in bone marrow were divided into three subgroups, one associated with hematopoietic stem cells, one performing the function of platelet biosynthesis, and one with the ability to promote immune response [15]. Circulating megakaryocytes originating from extrapulmonary tissues such as bone marrow and splenic sinuses are retained at the bifurcation of pulmonary vessels, and these megakaryocytes release single platelets in the pulmonary circulation by forming pseudopodia extensions. Whereas another class of resident megakaryocytes is present in the pulmonary interstitium, transcriptomics has shown that they are enriched in transcriptional signatures of immune-related processes, including pathogen recognition, phagocytosis, and antigen presentation [16]. Single-cell sequencing results showed that early immature megakaryocytes could also express immunoreactive genes, suggesting two possibilities for megakaryocytes to grow into immune megakaryocytes and thrombopoietic megakaryocytes [17]. At the same time, platelets from septic patients have transcriptional components such as platelet-associated histones that differ from normal platelets. The possible reason is that immune megakaryocytes proliferate and produce pathogenic platelets, which in turn express high levels of inflammatory response genes [18]. Additionally, lung transplantation was performed after lung perfusion in a mouse model lacking platelets and HSCs, and the platelet count and trilineage hematopoietic function of the mice returned to normal several months after transplantation. These results suggest that megakaryocytes and hematopoietic progenitor cells in the pulmonary interstitium can migrate into the bone marrow and restore hematopoietic dysfunction in the bone marrow [4].

## 3. Megakaryocytes and platelets are associated with lung injury

### 3.1. Acute lung injury

**Figure 2.**
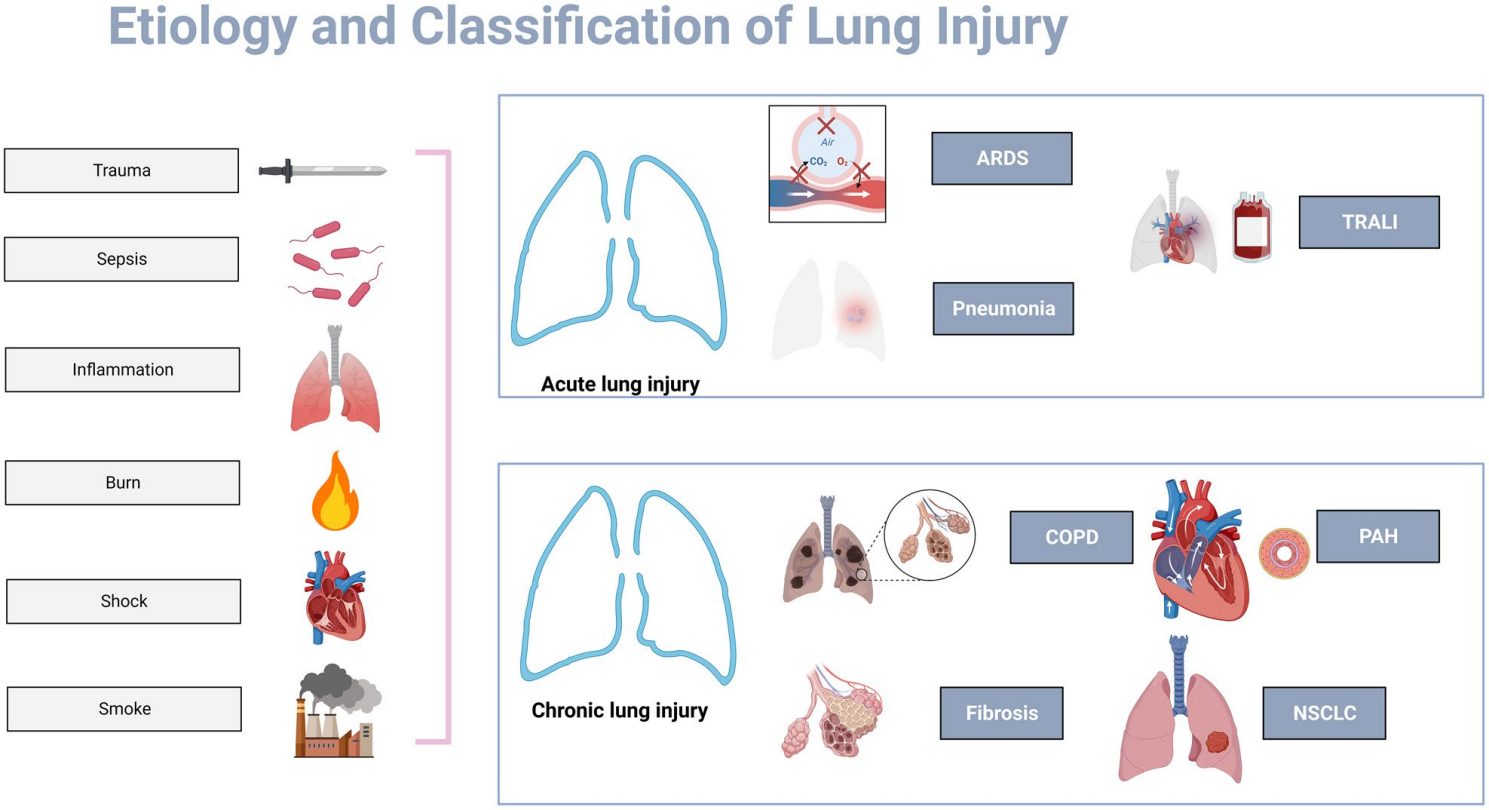
Etiology and Classification of Lung Injury. Trauma, sepsis, inflammation, burn, shock and smoke constitute the common causes of lung injuries. ARDS, pneumonia and TRALI are types of ALI with megakaryocytes and platelets involvement. Megakaryocytes and platelets also associated with chronic lung injury, e.g. COPD, PAH, lung fibrosis and NSCLC.

Acute lung injury (ALI) is acute hypoxic respiratory insufficiency or respiratory failure caused by injury factors such as trauma, burns, shock and severe infection that act on the body for a short time, causing alveolar epithelial cell and pulmonary capillary endothelial cell injury resulting in diffuse pulmonary interstitial and alveolar edema [19]. ALI can result from both direct injury (intrapulmonary factors) and indirect injury (extrapulmonary factors). Direct injury includes severe lung infection, inhalation lung injury, lung contusion, etc. Indirect injury includes sepsis, DIC, blood transfusion, burn, shock, etc [20]. In general, the mechanism of injury in ALI is divided into two parts: gas exchange disorder and inflammatory injury (Figure 2).

As a reservoir of platelet precursor (megakaryocytes), the lung is closely associated with platelets. Platelets not only play a role in hemostasis, regulating the pulmonary vascular barrier and lung tissue repair, but also act as inflammatory cells to stimulate non-hemostatic immune function and participate in the progression of various inflammatory diseases, leading to tissue damage. Therefore, it is speculated that megakaryocytes and platelets may play an important role in the pathogenesis of ALI [5].

#### 3.1.1. Mechanisms of platelet involvement in ALI

Studies have shown that platelets, on the one hand, limit the extravascular transport of water, protein, red blood cells, etc. as well as maintain and repair the barrier integrity of pulmonary vessels; on the other hand, platelets are involved in the progression of ALI and acute respiratory distress syndrome (ARDS) by regulating alveolar capillary permeability and interacting with neutrophils and macrophages [21]. Platelets participate in ALI through three mechanisms: immune regulation of platelet secretion, receptor-ligand signaling pathways related to platelet activation, and direct interactions between platelets and associated cells. First, platelets in small pulmonary vessels are activated at ALI, secrete platelet microparticles (PMPs), activate neutrophils and release proinflammatory factors such as proteases, IL-1, and tumor necrosis factor (TNF), which causes tissue damage. PMPs also promote transcellular transport of arachidonic acid and upregulates the expression of endothelial cyclooxygenase 2 and intercellular adhesion molecule 1 (ICAM-1), resulting in increased permeability of endothelial and alveolar epithelial cells and aggravating ALI [22]. Additionally, in a murine model of acid-induced ALI, platelets secrete thromboxane A2 (TXA2) when ALI occurs, which promotes platelet-neutrophil aggregation and also aggravates ALI injury [23]. Second, when ALI occurs, platelets may be activated *via* the receptor-ligand signaling pathway. Immunoreceptor tyrosine‑based activation motif (ITAM) receptor signaling pathway is essential for platelet activation by extracellular matrix and extravascular cells [24]. C-type lectin receptor 2 (CLEC‑2) is an ITAM receptor highly expressed on megakaryocytes and platelets and expressed at low levels on neutrophils in peripheral blood stream, which mediate endocytosis and release of pro-inflammatory factors such as TNF‑α. CLEC‑2 activates platelets *via* the tyrosine kinase signaling pathway, whereas the immunoglobulin-like receptor glycoprotein VI interacts with ligand collagen to activate platelets *via* the splenic tyrosine kinase pathway in mice [25]. At the same time, the combination of TXA2 to G protein-coupled thromboxane receptors can lead to a wide range of responses such as integrin activation, platelet aggregation, smooth muscle cell contraction, and increased vascular permeability, which are involved in the development of ALI [5]. Finally, platelets are activated to exert biological effects by direct contact with other cells. In lipopolysaccharide (LPS) -induced ALI mice model, Toll-like receptors (TLR) on the platelet surface are activated, resulting in the increase of mitochondrial reactive oxygen species (mtROS) production and leading to increased expression of CD62P and GPIIb/IIIa [26]. CD62P is a key ligand for platelet binding to neutrophils and monocytes and leads to the production of neutrophil extracellular traps (NETs) in ALI. NETs itself acts as a scaffold for platelet aggregation and participates in the coagulation cascade, whereas histones and neutrophil granule proteins in NETs damage endothelial and epithelial cells and participate in the progression of ALI [27]. Platelets can also mediate endothelial cell inflammatory responses through interaction with P-selectin-expressing endothelial cells *via* the integrin receptor GPIb/IX/V [28]. It has been shown that FcγRIIA on platelets binds IgG complexes and releases large amounts of sCD40L and RANTES, whereas sCD40L (Soluble CD40 ligand) directly contacts the CD40/CD40L complex to interact with T cells and promote platelet inflammation [29]. In addition, platelets interact with B cells *via* the Pattern Recognition Receptor (PRR) and undergo inflammatory signaling and apoptosis induction upon PRR-mediated cell activation and are involved in the progression of ALI [30]. In addition, acute hypoxia caused by ALI significantly increased the number of immature platelets and significantly elevated the extent and rate of ADP-induced platelet aggregation. In contrast, chronic hypoxia mainly caused an increase in the number of mature and senescent platelets, but the functional activity of platelets was lower than those in acute hypoxia [31].

#### 3.1.2. Mechanisms of megakaryocytes involved in ALI

Megakaryocytes in the lung not only have thrombopoietic function, but also express a series of genes related to pathogen recognition, inflammatory response, and innate immune response such as chemokines and inflammatory factors, which are involved in the protection or progression of ALI. Megakaryocytes also express major histocompatibility complex (MHC) class II, antigen presenting cell (APC) markers, dendritic cell markers, including CD11c, CD40, CD80, ICAM-1, and CC chemokine receptor 7 (CCR) [32]. Compared with megakaryocytes in the bone marrow, lung megakaryocytes express higher levels of TLR and CLEC and have the ability to sense as well as ingest pathogens, such as phagocytosis or uptake of the virus SARS-CoV-2 *in vivo*, which have been shown as as protection against ALI resulting from infection [33]. Lung megakaryocytes, unlike megakaryocytes in the bone marrow, were found to have increased expression of MHC II and ICAM 1 under the regulation of the lung-related immune molecule IL-33, exhibiting the characteristics of APC which internalizes and processes DQ -ovalbumin antigen and intact live E.coli both *in vitro* and *in vivo*. Meanwhile, lung megakaryocytes induce the activation of CD4 + T cells in an MHC II dependent manner both *in vitro* and *in vivo* [16]. The immunophenotypic difference between lung and bone marrow megakaryocytes may be caused by the communication between lung and external environment and the susceptibility to pathogen infection. Megakaryocytes in mice with high chemokine (C-X-C motif) receptor 4 expression have been shown to attenuate infectious factors in ALI by enhancing macrophage and neutrophil migration and bacterial phagocytosis through TNF-α and IL-6 production [34]. Lung megakaryocytes play an important role in resistance to pathogen invasion, but may also exacerbate ALI progression. Lung megakaryocytes can produce inflammatory factors such as chemokine (C-X-C motif) ligand 1 (CXCL1), chemokine (CC motif) ligand 2 (CCL-2), ICAM-1, IL-1α, SDF-1, macrophage inflammatory protein 3 (MIP-3), and TNF-α to promote ALI inflammatory response [16]. Acute hypoxia resulting from gas exchange impairment at ALI accelerates the transition of promegakaryocytes to mature megakaryocytes and increases megakaryocyte size. Chronic hypoxia, on the other hand, increases the number and size of megakaryocytes by increasing the production of megakaryocytic progenitors [31].

#### 3.1.3. Relationship between megakaryocytes and platelets and clinical diseases

In lung hemorrhagic injury such as lung trauma, platelets participate in the formation of initial thrombi and maintain pulmonary capillary endothelial integrity and barrier function by releasing factors such as S1P, serotonin (5-HT), and reduce pulmonary edema [35]. At the same time, platelets recruit vascular progenitor cells and precursor cells by releasing SDF-1α, integrins, P-selectin, and induce functional changes at sites of pulmonary thrombosis, pulmonary vascular injury, and ischemia, which in turn play a role in angiogenesis and development, vascular repair, and remodeling [36].

ARDS is a progressive stage of ALI in which pulmonary microvascular injury renders megakaryocytes unable to lyse into platelets in the lung and thus increase in number. In parallel, platelets are largely consumed under inflammatory conditions, and expression of TPO is elevated, thereby stimulating megakaryocyte differentiation in the bone marrow and circulation to the lung [37]. However, the increased number of lung megakaryocytes will promote platelet replenishment, promote neutrophil migration to inflammatory sites through platelet secretion of inflammatory factors, and accelerate the development of ARDS. In addition, triggering receptor expressed on myeloid cells-like (TREM-like) transcript-1 (TLT-1) on platelets deposits fibrinogen on inflammatory tissue and guides neutrophil migration, inhibiting the inflammatory response early in ARDS. Whereas in the late stage of ARDS, soluble TLT-1 fragments promote platelet aggregation and adhere to fibrinogen, which cause abnormal fibrinogen deposition leading to DIC [38]. Thus, lung megakaryocytes accelerate the progression of ARDS by replenishing platelet production, while antiplatelet therapy has a favorable effect on improving ARDS prognosis. Studies have shown that hypoxia-inducible factor-1 (HIF-1) is activated in response to ARDS and promotes granular alveolar cell proliferation and migration through vascular endothelial growth factor (VEGF) and SDF1 signaling, which facilitate alveolar epithelium repair [39]. In parallel, reduced expression of HIF-1α may lead to impaired megakaryopoiesis in mouse bone marrow cells treated with immune thrombocytopenia (ITP) plasma [40]. It is hypothesized that the HIF-1 signaling pathway also plays an important role in platelets and megakaryocytes when ARDS occurs.

Lung megakaryocytes and platelets play distinct roles in pulmonary infectious injury. Klebsiella pneumoniae infection leads to platelet aggregation and apoptosis, and inhibits megakaryocyte maturation, thereby significantly reducing the number of platelets and increasing the risk of bleeding and mortality in mouse infection models [41]. In addition, when human body is infected with SARS-COV-2, the massive consumption of platelets and the massive expression of inflammatory factors such as IL-6, interferon A (IFN-A), and serum C-reactive protein (CRP) promote the production of pulmonary megakaryocytes [42]. Pulmonary megakaryocytes, on the other hand, improve antiviral capacity in humans by enhancing the regulation of type 1 IFN response, and increase PLT aggregation to promote coagulation [43]. Meanwhile, lung megakaryocytes express immune response-related genes such as TLR3, which recognizes double-stranded RNA (dsRNA) associated with viral infection to enhance platelet function and reduce platelet production [44]. Therefore, promoting pulmonary megakaryopoiesis or improving pulmonary megakaryocyte function may serve as a treatment for COVID-19. Thrombin-activated platelets have been shown to cause lung injury when influenza A virus infects the lung by recruiting neutrophils and promoting NETs release. Antagonism of protease-activated receptor 4 (the main thrombin receptor) on platelets or inhibition of thrombin activation by antithrombin III provides a novel target for protection against infectious lung injury [45]. In sepsis secondary to burn injury, megakaryocytes exhibit multiple immune cell functions such as chemotaxis, interaction with pathogens, and release of histone-modified chromatin networks [46]. Platelets activated by LPS, on the other hand, promote the formation of NETs *via* autocrine 5-HT and aggravate sepsis induced ALI [47].

Platelet transfusion is one of the causes of transfusion-related acute lung injury (TRALI). Platelet transfusion plays a dual role in the development of TRALI. Thrombin-activated platelets can accumulate into microvascular thrombi *via* GPIIb/IIIa, or recruit neutrophils *via* P-selectin, inducing the formation of NET, thereby promoting inflammatory responses and tissue damage [48]. Activated platelets may also aggravate TRALI by releasing cytokines such as IL-1β and TNF-α that interfere with the stability of endothelial barrier function. In contrast, resting platelets bind neutrophils *via* GPIb and endothelial cells *via* GPVI to protect against pulmonary edema [49].

### 3.2. Chronic lung injury

Lung megakaryocytes and platelets are implicated in lung growth and repair. Lung megakaryocytes have growth factors such as SDF-1, transforming growth factor-β (TGF-β), and insulin-like growth factor-1 (IGF-1) that are essential for alveolar formation and development. CLEC-2 produced by platelets stimulates alveolar duct myofibroblast differentiation of pulmonary mesothelial cells *via* TGF-β signaling, thereby regulating normal lung growth [50]. However, platelet-derived SDF-1 stimulates the expression of SDF-1 receptor on pulmonary capillary endothelial cells and enhances alveolar epithelial cell proliferation and lung regeneration. Excessive tissue repair responses following chronic lung injury may lead to fibrosis. In a mouse model of bleomycin-induced lung injury, thrombopoietin (TPO) activates megakaryocytes *via* TGF-β and promotes fibroblast proliferation and transdifferentiation into myofibroblasts, leading to pulmonary fibrosis [51]. Chronic hypoxia resulting from chronic lung injury may cause thrombocytopenia while concurrently causing erythrocytosis in individuals, which is associated with a prolonged hypoxic environment that inhibits megakaryocyte differentiation and maturation and promotes platelet apoptosis [52].

Chronic obstructive pulmonary disease (COPD) usually results from chronic damage to the airways or pulmonary bronchi by toxic particles or gases. Exposure to fine particulate matter (PM) in air significantly increased DNA ploidy and promoted maturation of megakaryocytes in an exposure-dependent manner. At the same time, megakaryocytes became larger under PM exposure and megakaryocytes produced bud-like projections that favored thrombopoiesis. Proteomic studies have shown that PM promotes megakaryocyte maturation and thrombopoiesis mainly through regulating mitochondrial oxidative phosphorylation, producing reactive oxygen species (ROS) to induce oxidative stress [53], which increase the risk of thrombosis in COPD patients. Therefore, COPD can be clinically intervened from the perspective of PM regulation of megakaryocyte thrombopoiesis as well as mitochondrial oxidative phosphorylation.

Lung megakaryocytes contain various pulmonary fibrosis-related growth factors such as FGF, TGF, and platelet-derived growth factor (PDGF), and neutrophils induce the release of these growth factors from megakaryocytes by binding to P-selectin on megakaryocytes and induce the production of collagen and reticulin [54,55]^.^ Lysyl oxidase (LOX) in lung megakaryocytes cross-links collagen and elastin, promotes extracellular matrix accumulation, and aggravates pulmonary fibrosis [56]. Recently, 5-HT in platelets has been found to be a mediator linking vascular injury and extravascular tissue fibrosis, indicating that 5-HT signaling pathway is a new clinical therapeutic target for pulmonary fibrosis [57], which also provides ideas for the treatment of later pulmonary fibrosis in patients with burn complicated by inhalation lung injury. At the same time, mean platelet volume and alterations in a blood megakaryocyte gene signature may have prognostic value in idiopathic pulmonary fibrosis (IPF) [58].

The differences in morphology and number of the lungs megakaryocytes were found in patients with idiopathic pulmonary arterial hypertension (IPAH), suggesting an influencing factor in the pulmonary circulation [59]. In chronic pulmonary arterial hypertension (PAH) disease, platelet mitogens, including 5-HT and PDGF, may cause pulmonary vascular smooth muscle cell proliferation in patients [60]. In a mouse model of hypoxia, deletion of Toll-like receptor 4 in platelets decreased plasma 5-HT levels as well as pulmonary arteriolar muscularization, right ventricular hypertrophy, and pulmonary arterial pressure [61]. In PAH, platelet metabolism shifts toward aerobic glycolysis with enhanced basal glycolytic rate and mitochondrial respiratory reserve [62]. Whereas megakaryocytes exhibit enhanced thrombopoiesis under hyperoxic or prooxidative conditions. Similarly, mtROS induces megakaryocyte maturation and promotes platelet production [63]. This suggests new ideas for mitochondrial therapy of platelets in PAH progression and therapeutic response.

Lung megakaryocytes and platelets are closely related to the development of lung cancer. Studies have shown that increased numbers and densities of megakaryocytes in tissues from non-small cell lung cancer (NSCLC) are associated with worse prognosis. Because lung blood supply is present in lung cancer tissues and SDF-1 is often overexpressed in NSCLC tissues, chemotaxis of megakaryocytes in the pulmonary circulation occurs. Megakaryocytes that migrate to the blood vessels of lung cancer tissue produce more platelets, helping tumor cells break through the endothelial barrier to achieve transendothelial migration and thus promoting the development and metastasis of lung cancer [64]. In early stage of lung cancer, increased expression of thrombospondin-1 (TSP-1) in lung megakaryocytes and platelets inhibits endogenous angiogenesis and exerts antitumor effects [65]. However, pulmonary megakaryocytes secrete platelet factor 4 (PF4), which in turn promotes hematopoietic function and megakaryocyte production under stress conditions caused by chemotherapy and radiotherapy [66], accelerating tumor growth. Megakaryocyte-generated platelets encapsulate tumor cells and protect them from cytotoxic effects as well as TNF and natural killer cell-mediated immune system attack [67]. In conclusion, regulation of megakaryocyte and platelet function in the tumor microenvironment can play a therapeutically regulatory role in NSCLC.

## 4. Prospects

Platelets in the lung are derived not only from the spleen and bone marrow, but also from megakaryocytes in the pulmonary circulation. Current studies have shown that pulmonary megakaryocytes not only produce platelets to play a hemostatic role in lung injury, but also participate in inflammation and immune response with platelets to promote the process of lung injury or play a protective role. Therefore, targeting lung megakaryocytes and platelets is currently a new direction for the treatment of a variety of lung injuries. Studies have shown that pegylated conjugated TPO and thrombopoietin receptor agonists such as eltrombopag and altrombopag specifically stimulate megakaryopoiesis and thrombopoiesis, which present a significant therapeutic effect on thrombocytopenia after chemotherapy for lung cancer [68]. Platelet-derived TLT-1 reflects the degree of lung injury and is an independent prognostic indicator of ALI and ARDS [38], whereas antiplatelet therapy has been shown to have a significant effect in reducing the incidence of ARDS [69]. In COPD, platelets secrete PF4 to break down pulmonary elastin, causing emphysema; upon vascular injury or hypoxic stress, platelets activate to aggregate and lead to pulmonary vascular remodeling, subsequently causing pulmonary hypertension (PH). Antiplatelet therapy with aspirin or clopidogrel effectively improved the symptoms of COPD and reduced their mortality [70]. At the same time, mitrozine treats lung injury by reducing oxidative stress and mitochondrial damage under hypoxia by acting on platelet-type phosphofructokinase to regulate glycolysis [71]. In the treatment of sepsis secondary to burn injury, turmeric, a traditional Chinese medicine, ameliorates sepsis-induced lung injury by inhibiting platelet-mediated NETs formation [72]. It has been shown that pulmonary megakaryocytes promote the generation of microthrombi, induce the generation of new blood vessels and cause pulmonary fibrosis at COVID-19 [54]. Intervention against pulmonary megakaryocytes is a new direction for relevant drug development. In addition, as an important factor in NSCLC progression, mRNA, ncRNA, lipids, proteins in platelets and their extracellular vesicles can serve as biomarkers and play a role in the diagnosis and prognostic evaluation of NSCLC [73]. Combined with swarm intelligence, tumor-educated blood platelets (TEPs) also provide a non-invasive detection method of NSCLC [74]. In conclusion, it is of great significance to explore the novel mechanism of megakaryocytes and platelets acting on lung injury for the diagnosis and treatment of lung injury.

## Data Availability

The references used to support the findings of this study are included within the article. Figures were created with BioRender.com.
